# Prescription Drug Promotion from 2001-2014: Data from the U.S. Food and Drug Administration

**DOI:** 10.1371/journal.pone.0155035

**Published:** 2016-05-05

**Authors:** Helen W. Sullivan, Kathryn J. Aikin, Eunice Chung-Davies, Michael Wade

**Affiliations:** U.S. Food and Drug Administration, Silver Spring, Maryland, United States of America; University of North Carolina at Charlotte, UNITED STATES

## Abstract

The volume of prescription drug promotion over time is often measured by assessing changes in ad spending. However, this method obscures the fact that some types of advertising are more expensive than others. Another way to measure the changes in prescription drug promotion over time is to assess the number of promotional pieces submitted to the U.S. Food and Drug Administration (FDA). Form FDA 2253 collects information such as the date submitted and the type of material submitted. We analyzed data from Forms FDA 2253 received from 2001–2014. We examined the frequency of submissions by audience (consumer and healthcare professional) and type of promotional material. There was a noted increase in prescription drug promotion submissions across all media in the early 2000s. Although non-Internet promotion submissions have since plateaued, Internet promotion continued to increase. These results can help public health advocates and regulators focus attention and resources.

## Introduction

Advertising of prescription drugs to consumers and healthcare professionals can impact important health outcomes, such as diagnosis and treatment.[1–4] The volume of prescription drug promotion over time is often measured by assessing changes in ad spending.[5–8] However, this method obscures the fact that some types of advertising (e.g., television ad campaigns) are more expensive than others (e.g., brand websites). Another way to measure the changes in prescription drug promotion over time is to assess the number of promotional pieces submitted to the U.S. Food and Drug Administration (FDA). FDA regulations state that prescription drug advertising and promotional labeling must be submitted when it is first disseminated.[9–10] FDA uses Form FDA 2253 to accompany this information.[11] Information from this form can be used to examine the type and amount of the many promotional materials submitted to FDA over time. The objective of the current study was to determine the type of prescription drug promotional materials submitted to FDA from 2001–2014 in terms of the audience (consumer and healthcare professional) and media type (e.g., print versus television).

## Materials and Methods

Form FDA 2253 collects information such as the date submitted and the type of material submitted. FDA uses codes to classify the promotional materials submitted on Form FDA 2253 into categories (e.g., television ad). Some of these codes have changed over time. Notably, Internet promotion (including websites, web videos, web audio, sponsored links, social media, mobile applications, and emails) was not categorized separately for consumers and healthcare professionals until 2011.

**Fig 1:**
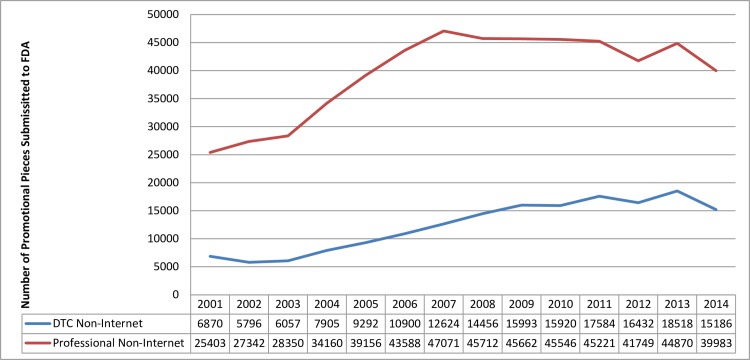
Non-internet promotion.

**Fig 2:**
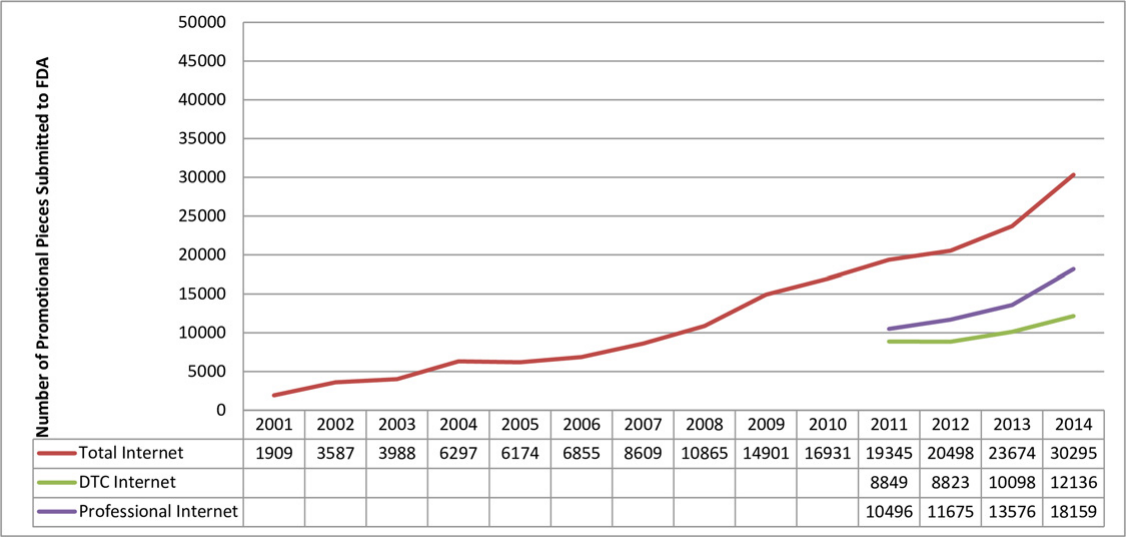
Internet promotion. *Note*. Internet promotion was not categorized separately for consumers and healthcare professionals until 2011.

**Fig 3:**
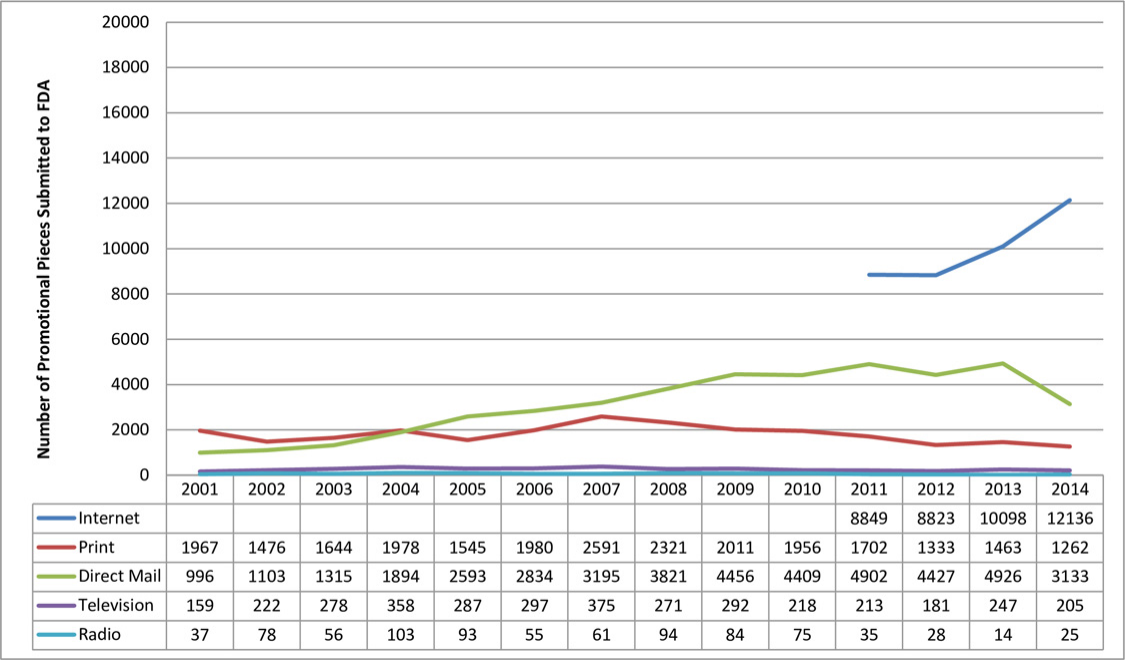
Direct-to-consumer promotion. *Note*. The print category includes print ads, brochures, books, and reply cards.

**Fig 4:**
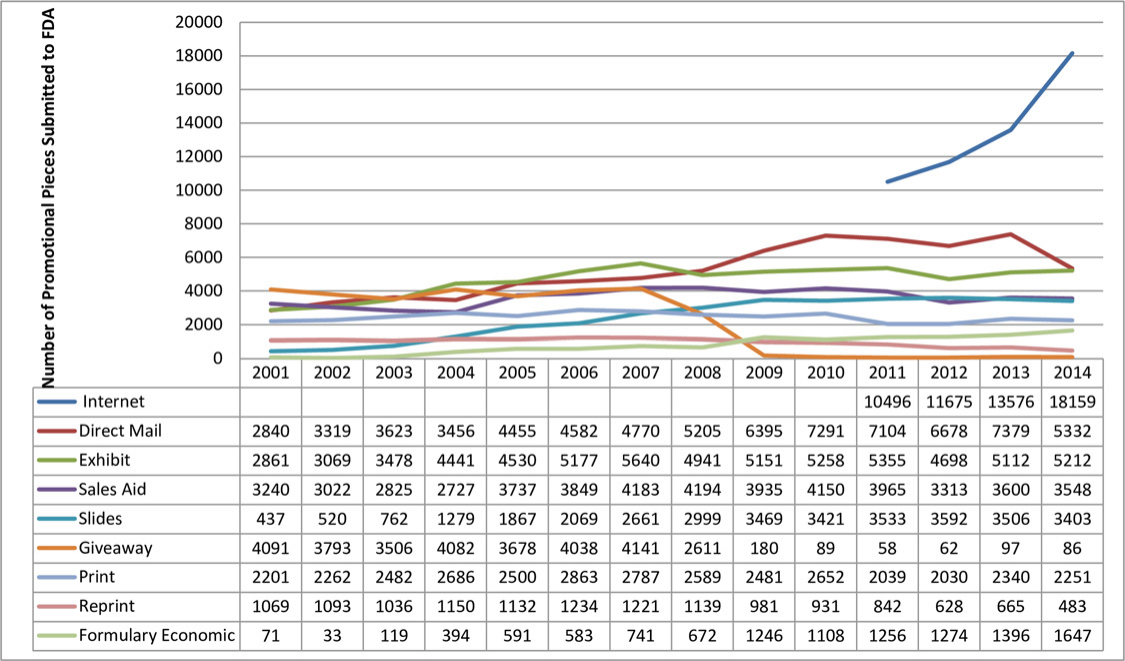
Professional promotion. *Note*. The print category includes print ads, brochures, books, and reply cards.

We analyzed data from Forms FDA 2253 received from 2001–2014. We examined the frequency of submissions by audience (consumer and healthcare professional) and type of promotional material (Figs 1 and 2). For a more in-depth look at the types of promotion submitted to FDA, we examined categories that had over 10,000 pieces from 2001–2014 (Figs 3 and 4). For direct-to-consumer (DTC) promotion, we also included television and radio for comparison to ad spending data.[7] Although the “other/unknown” category accounted for the largest number of DTC and professional pieces submitted, we excluded these categories from Fig 3 and Fig 4 to focus on more informative categories. To create an overall category for print promotion across audiences, we combined print ads, brochures, books, and reply cards.

## Results

FDA received approximately three times as many non-Internet submissions directed toward healthcare professionals as submissions directed toward consumers from 2001–2014 (Fig 1). This gap was smaller for Internet submissions, at approximately 1.5 times as many healthcare professional submissions for 2011–2014 (Fig 2). Submissions for non-Internet promotion directed toward consumers increased from 6,870 in 2001 to 15,993 in 2009, and then plateaued, with 15,186 in 2014. Non-Internet promotion directed toward healthcare professionals shows a similar trend: submissions increased from 25,378 in 2001 to 47,071 in 2007, and then plateaued, with 39,983 in 2014 (Fig 1). In contrast, submissions for Internet promotion steadily increased from 2001–2014 (Fig 2). In fact, from 2011–2014 (the years during which Internet promotion submissions were separated by audience) Internet promotion was the single largest category of FDA Form 2253 submissions for both consumer and healthcare professionals (excluding the “other/unknown” category; Figs 3 and 4).

Internet, direct mail (printed non-electronic materials mailed directly to individuals), and print ads were the largest categories of DTC promotion submitted on FDA Form 2253, with television and radio promotion comprising only a small percentage of submissions (Fig 3). As with DTC promotion, Internet, direct mail, and print ads were also large sources of professional promotion submitted on FDA Form 2253 (Fig 4). Office-, hospital-, and conference-based promotion accounts for a large percentage of professional promotion submitted on FDA Form 2253, including exhibits, sales aids, slides, reprints, and formulary economic (material containing cost information about a product provided to a formulary committee). In addition, the effects of self-imposed industry guidelines can be seen in Fig 4: the number of submissions for giveaways to healthcare professionals dropped dramatically (from 4,141 in 2007 to 86 in 2014) after self-imposed industry guidelines issued in 2008 prohibited the use of non-educational giveaways.[12]

## Discussion

This study allowed a look at a previously unpublished source of data: submission of prescription drug promotional materials to FDA. The results show that FDA receives tens of thousands of promotional materials every year. This corresponds with the high level of exposure to DTC advertising that individuals reported during this time period.[13–14] FDA conducts surveillance in a variety of ways: through surveillance at medical conventions, review of promotional pieces submitted via FDA Form 2253, and review of submissions of complaints.[15] FDA may not be able to review every submission because of the sheer volume; however, these data can help FDA allocate resources more efficiently.

A number of trends emerged over time, including the turn to an increase in promotion via the Internet. Similarly, a recent analysis of DTC ad spending found that only Internet-based DTC advertising showed substantial growth from 2005–2009.[8] A study published in 2007 identified 91 prescription drug websites, [16] and a 2010 analysis of the top 10 selling drugs found that 9 had a prescription drug website, 9 had Twitter/Friendster traffic, 8 were promoted on YouTube, and 7 had Facebook pages.[17] The shift in focus to Internet-based prescription drug promotion may reflect both economic pressures on the industry and a practical focus on the needs of the audience. According to a 2012 survey, 72 percent of adults used the Internet to search for health information,[18] and the Internet is often the first place people look for health information.[19] In a recent survey, 12% of adults reported that they visit pharmaceutical company websites for information regarding their healthcare needs, and 37% reported that an advertisement might motivate them to visit a pharmaceutical company website.[20] It makes sense, then, for ads to follow their audience online. The non-linear structure of the Internet facilitates new approaches to prescription drug promotion. These innovative promotional options provide a challenge to implementing the regulations. Accordingly, regulators may need to further contemplate Internet-based promotional activities.

Although attention is often focused on DTC advertising, these results and ad spending data demonstrate that the majority of prescription drug promotion continues to be targeted toward healthcare professionals.[7] Research demonstrates that healthcare professionals are influenced by drug promotion, even if they do not believe they are.[4,21] Thus, attention to the impact of professional advertising continues to be warranted. The trends that emerged in the analysis of FDA Form 2253 data both reflected and complemented findings from analyses of ad spending data. For instance, although these results and ad spending data show an increase in Internet promotion over time, the ad spending data indicate that Internet promotion is a fraction of ad spending. In contrast, Internet promotion accounts for a large percentage of FDA Form 2253 submissions. The reverse is true for television promotion: it accounts for a large percentage of ad spending but a small percentage of FDA Form 2253 submissions. While ad spending data show an increase in spending until 2004 for healthcare professionals and 2006 for DTC,[5,7] the increase in FDA submissions lasted three years longer (2007 for healthcare professionals and 2009 for DTC). This may reflect a lag between spending on ad development and ad dissemination or use.

Analyses of spending data are limited because they do not account for unequal costs across media and may obscure changes in the ability to target ads to particular audiences. The main limitation of analyzing the submissions to FDA is that it does not take into account the frequency of the advertising. For instance, this method allows us to say that a television ad was submitted to FDA, but not whether or how many times it aired on television. Therefore, it is complementary to the assessment of ad spending and other methods, such as directly assessing the number of television ads aired.[3, 22, 23] Another possible limitation is that analyzing the submissions to FDA may not capture all promotion because a pharmaceutical company may not comply with the regulations; however, a search of FDA’s enforcement letters suggests that this is not a common occurrence.

These data provide a window into the shifting focus of prescription drug promotion over time. Understanding the audience and media for this type of promotion can assist public health advocates and regulators to target policy development and allocate resources based on data.

## References

[1] Gilbody S, Wilson P, Watt I. Benefits and harms of direct to consumer advertising: A systematic review. Qual Saf Health Care. 2005;14(4):246–250. 16076787 10.1136/qshc.2004.012781 PMC1744049

[2] Mintzes B. Advertising of prescription-only medicines to the public: Does evidence of benefit counterbalance harm? Annu Rev Public Health. 2012;33:259–277. 10.1146/annurev-publhealth-031811-124540 22429162

[3] Niederdeppe J, Byrne S, Avery RJ, Cantor J. Direct-to-consumer television advertising exposure, diagnosis with high cholesterol, and statin use. J Gen Intern Med. 2013;28(7):886–893. 10.1007/s11606-013-2379-3 23463454 PMC3682042

[4] Spurling GK, Mansfield PR, Montgomery BD, Lexchin J, Doust J, Othman N, et al Information from pharmaceutical companies and the quality, quantity, and cost of physicians' prescribing: A systematic review. PLoS Med. 2010; 7(10):e1000352 10.1371/journal.pmed.1000352 20976098 PMC2957394

[5] Bulik BS. Ad spending: 15 years of DTC. Pharmaceutical marketing. Ad Age. [Internet]. 2011 [cited 2016 Jan 8]. Available from: http://adage.com/images/bin/pdf/WPpharmmarketing_revise.pdf

[6] Donohue JM, Cevasco M, Rosenthal MB. A decade of direct-to-consumer advertising of prescription drugs. N Engl J Med. 2007;357:673–681. 17699817 10.1056/NEJMsa070502

[7] Kornfield R, Donohue J, Berndt ER, Alexander GC. Promotion of prescription drugs to consumers and providers, 2001–2010. PLOS ONE. 2013;8(3):e55504 10.1371/journal.pone.0055504 23469165 PMC3587616

[8] Mackey TK, Cuomo RE, Liang BA. The rise of digital direct-to-consumer advertising?: Comparison of direct-to-consumer advertising expenditure trends form publicly available data sources and global policy implications. BMC Health Serv Res. 2015; 15:236 10.1186/s12913-015-0885-1 26084705 PMC4472256

[9] Other postmarketing reports: Other reporting–advertisements and promotional labeling, 21 C.F.R Sect. 314.81(b)(3)(i) (2014).

[10] Changes to an approved application: Labeling changes–advertisements and promotional labeling, 21 C.F.R. Sect. 601.12(f)(4) (2014).

[11] U.S. Food and Drug Administration. OPDP Form FDA 2253 and Request for Advisory Comment Submissions [Internet]. 2015 [cited 2016 Jan 8]. Available from: http://www.fda.gov/AboutFDA/CentersOffices/OfficeofMedicalProductsandTobacco/CDER/ucm448915.htm

[12] Pharmaceutical Research and Manufacturers of America. Code on Interactions with Healthcare Professionals [Internet] 2008. [cited 2016 Jan 8]. Available from: http://www.phrma.org/sites/default/files/pdf/phrma_marketing_code_2008-1.pdf

[13] Alperstein NM. Awareness of and attitudes toward direct-to-consumer prescription drug advertising among young adults. Health Mark Q. 2014;31(3):231–245. 10.1080/07359683.2014.936291 25120044

[14] Krahn J. What are adults doing as a result of healthcare advertising? Kantar Media. [Internet] 2013. [cited 2016 Jan 8]. Available from: http://www.kantarmedia-healthcare.com/what-are-adults-doing-as-a-result-of-healthcare-advertising

[15] Abrams T. OPDP update on oversight of prescription drug promotion. Presented at: The Food and Drug Law Institute Advertising and Promotion Conference; 2012 October 1–2; Washington, D.C.

[16] Sheehan KB. Direct-to-consumer (DTC) branded drug web sites risk presentation and implications for public policy. J Advert. 2007;36(3):123–135.

[17] Liang BA, Mackey TK. Prevalence and global health implications of social media in direct to-consumer drug advertising. J Med Internet Res. 2001;13(3). 10.2196/jmir.1775 PMC3222189 21880574

[18] Pew Research Center. Health fact sheet [Internet]. 2015. [cited 2016 Jan 8]. Available from: http://www.pewinternet.org/fact-sheets/health-fact-sheet/

[19] Hesse BW, Moser RP, Rutten LJ. Surveys of physicians and electronic health information. N Engl J Med. 2010;362(9):859–860. 10.1056/NEJMc0909595 20200398

[20] Makovsky PR. Sixth Annual Makovsky/Kelton pulse of online search survey: Initial data. [Internet] 2016. [cited 2016 Apr 13]. Available from: http://www.makovsky.com/images/makovsky/downloads/makovsky_2016_health_survey.pdf

[21] Steinman MA, Shlipak MG, McPhee SJ. Of principles and pens: Attitudes and practices of medicine housestaff toward pharmaceutical industry promotions. Am J Med. 2001;110:551–557. 11347622 10.1016/s0002-9343(01)00660-x

[22] Brownfield ED, Bernhardt JM, Phan JL, Williams MV, Parker RM. Direct-to-consumer drug advertisements on network television: an exploration of quantity, frequency, and placement. J Health Comm. 2004;9(6):491–497. 10.1080/10810730490523115 15764448

[23] Kornfield R, Alexander GC, Qato DM, Kim Y, Hirsch JD, Emery SL. Trends in exposure to televised prescription drug advertising, 2003–2011. Am J Prev Med. 2015;48(5):575–579. 10.1016/j.amepre.2014.12.001 25891057 PMC4405658

